# Impact of excessive environmental information disclosure on stock price crash risk

**DOI:** 10.1371/journal.pone.0338808

**Published:** 2025-12-23

**Authors:** Guoping Dong, Guifen Ma

**Affiliations:** Accounting Institute, Guangzhou Huashang College, Guangzhou, China; Sri Lanka Institute of Information Technology - Malabe Campus: Sri Lanka Institute of Information Technology, SRI LANKA

## Abstract

With the deepening of carbon peak and carbon neutrality (“dual carbon”) initiatives, corporate responsibility for environmental information disclosure has become imperative. However, due to imperfect laws and regulations, companies may have incentives to over-disclose environmental information, which could trigger stock price crashes. This study investigates the behavior of excessive environmental information disclosure among A-share listed companies in China. Using a sample of A-share firms that published social responsibility reports from 2015 to 2023, we employ threshold effect and quantile regression models to verify the presence of “greenwashing” components in environmental textual disclosures. A panel fixed-effects model is further adopted to examine the potential impact of excessive environmental information disclosure on stock price crash risk. The findings reveal that corporate environmental disclosures contain non-substantive, embellished content-indicative of greenwashing-and that such behavior significantly exacerbates stock price crash risk, particularly in manufacturing industries. The mechanism lies in the fact that excessive textual disclosure reduces information quality and transparency, thereby amplifying irrational investment behaviors. Conversely, effective environmental disclosure practices are shown to mitigate crash risk. Further analysis demonstrates that reducing ownership concentration, increasing managerial shareholding, and enhancing the role of independent directors in corporate governance can improve the quality of environmental disclosure and curb over-disclosure. This study provides a novel analytical perspective on environmental textual disclosure and offers practical insights for guiding rational investor decision-making.

## 1. Introduction

The 20th National Congress of the Communist Party of China clearly emphasized that ecological environmental protection is an essential component of Chinese-style modernization and encouraged social capital to engage in green and low-carbon technological innovation. As major consumers of energy and significant polluters [1], companies’ environmental information disclosure is a crucial aspect of their social responsibility and involvement in ecological governance. Accurate and comprehensive environmental disclosures play an important role in addressing pollution prevention and promoting modernized ecological governance. In recent years, the environmental information disclosed by Chinese companies has shown considerable heterogeneity, which can be attributed to several key factors. Firstly, the lack of a unified standard for the information disclosure framework [2] and unclear boundaries [3] contribute to this issue. Secondly, there are no clear restrictions on the disclosure matters or textual disclosure requirements, giving some companies significant discretion in their reporting [4]. Thirdly, corporate social responsibility (CSR) reports are not required to be audited by independent third-party organizations. Collectively, these factors reflect the absence of rigorous regulatory oversight and the inefficiency of existing monitoring mechanisms. As the concepts of carbon peaking and carbon neutrality become more deeply ingrained, companies may exhibit opportunistic tendencies in their environmental disclosures-aiming to protect their reputation and mitigate environmental compliance risks [5]. The embellishment of environmental information and excessive greenwashing exacerbate information asymmetry, which not only hinders the development of a green economy but also sends misleading signals to the capital market. This can lead to irrational investment behavior and, ultimately, stock price crashes.

A stock price crash-the Waterloo of stock prices-occurs when management has long concealed negative information and, upon the accumulation of such news reaching a critical threshold, releases it all at once. This sudden disclosure delivers a fatal blow to the stock price, triggering the crash. Such a phenomenon not only damages the interests of minority shareholders but also pushes the company to the brink of collapse. Existing studies on the determinants of stock price crashes primarily focus on three aspects. First, from the perspective of principal–agent conflicts, researchers have examined the relationships between stock price crashes and factors such as the shareholding ratio of major shareholders [6], the exit threat of non‐controlling shareholders [7], overinvestment [8], investor protection [9], relationships with online investors [10], excessive executive compensation [11], managerial overconfidence [12,13], and management disclosures [14]. Second, from the perspective of independent directors’ characteristics, studies have investigated how factors such as media background [15], celebrity independent directors [16], geographic location [17], network position [18], and the compensation of financial independent directors [19] affect stock price crashes. Third, from the perspective of information asymmetry, research has explored the impact of internal controls [20] and financial reporting transparency [21] on the likelihood of a stock price crash.

Research on environmental information disclosure has predominantly been conducted along three dimensions: motivations, governance, and outcomes. With regard to motivations, scholars have primarily examined corporate greenwashing—defined as the strategic exaggeration of environmental disclosures to construct a spurious green image. Empirical evidence suggests that internal drivers of greenwashing include weaknesses in internal control mechanisms, insufficient environmental ethical awareness, and the pursuit of green premiums [22]. Externally, greenwashing is shaped by factors such as the regulatory framework for disclosure, the intensity of environmental regulation, and inconsistencies in market-based environmental ratings.

From a governance perspective, a variety of factors have been identified as constraints on corporate greenwashing, including the stage of organizational transformation [23], technological advancement [24], financial resources released by artificial intelligence applications [25], digital transformation initiatives [26], the level of green innovation [27], corporate performance [28], banking competition [29], the expansion of green finance [30], the development of financial technology [31], as well as external monitoring from media and analysts [32,33]. In addition, stronger government oversight has also been shown to play a critical role in curbing greenwashing practices, thereby increasing the difficulty for market participants in evaluating the quality of environmental information disclosure.

As for the effects of environmental information disclosure, there is no unified conclusion. On one hand, environmental information disclosure helps improve corporate value [34], innovation capacity [35], reduces the likelihood of stock price crashes [36], and lowers external financing costs [37]. Positive environmental information disclosure can also facilitate government subsidies for companies [38]. On the other hand, some companies engage in “greenwashing” [39], disguising operational issues [40]. This occurs when environmental information disclosures do not align with actual environmental behaviors [2,4], with reports often favoring positive outcomes while ignoring negative ones [41]. This indicates that companies may engage in pseudo-social responsibility behaviors [42], which not only reduce the quality of information disclosure and corporate profits but also exacerbate long-term risks [43]. Furthermore, it increases divergence in CSR report ratings by rating agencies [44], making it more challenging for market participants to assess the quality of environmental information disclosures.

While prior studies have explored various aspects of environmental information quality, no consensus has been reached on the quality of environmental disclosure itself, and research examining the effect of excessive textual embellishment on stock price crash risk remains absent. In China, where communication is highly context-dependent, qualitative language tends to be diverse, elastic, and subtle in meaning [45]. Coupled with the absence of a unified disclosure framework, firms often employ strategic textual descriptions—driven by incentives to reduce environmental management costs—that are prone to misleading investors’ interpretation of information. To address this gap, we build on Zhang [43] rategy information (e.g., strategies and policies) and environmental action information (e.g., pollution-control expenditures and utilization of environmental protection equipment) [4]. The dictionary contains 1,597 keywords. Using JAVA-based web crawling techniques, we collected 10,899 valid CSR reports issued by A-share listed firms between 2015 and 2023. After data cleaning, the final dataset consists of 6,966 firm-year observations from 1,053 firms. To identify textual embellishment in environmental disclosure, we apply both threshold effect models and quantile regression analyses. We then classify firms into two groups—excessive disclosure (above the median disclosure volume) and effective disclosure (below the median)—and examine the impact of excessive disclosure on stock price crash risk.

The findings of the study are as follows: (1) There is widespread greenwashing behavior in environmental information among the sample companies. (2) Excessive environmental embellishment exacerbates the risk of stock price crashes. In contrast, effective environmental disclosures have a mitigating effect on stock price crashes. Excessive greenwashing increases information asymmetry in the capital market, heightening the risk of stock price crashes. On the other hand, appropriate and effective environmental text disclosures serve to suppress the risk of stock price crashes, with this phenomenon being particularly pronounced in the manufacturing industry. The mechanism at play is that excessive textual disclosure reduces the quality and transparency of information, leading to irrational investor behavior. (3) Further analysis shows that reducing ownership concentration, increasing management stockholding, and promoting the active role of independent directors in corporate governance can effectively improve the quality of environmental text disclosures and suppress excessive environmental embellishment.

The contributions of this paper are primarily reflected in the following two aspects: Theoretically, it enriches the research on the role of environmental information disclosure quality by empirically analyzing how excessive textual disclosure of environmental information exacerbates stock price volatility, which is detrimental to stock price stability. Practically, environmental information embellishment serves as a tool in the strategic game between companies and market investors. Given that environmental information embellishment is prevalent in most corporate social responsibility (CSR) reports, this study alerts investors to enhance their ability to identify environmental information, emphasizing the importance of not only “listening to words but also observing actions.” At the same time, it provides valuable insights for regulators to optimize information screening mechanisms and improve the ecological system.

## 2. Theoretical analysis and research hypotheses

### 2.1. Existence of environmental text embellishment

With the deepening of the carbon peaking and carbon neutrality agenda, corporate stakeholders have increasingly paid attention to firms’ environmental information disclosure. However, in the absence of a fully developed disclosure framework, firms retain considerable discretion over the content of environmental textual information [4]. Since the promulgation of the Green Credit Guidelines in 2012, green credit has imposed environmental access thresholds on high-energy-consuming and high-polluting industries, while green finance has favored firms with a better environmental image. Moreover, due to compliance requirements and the relatively low cost of embellishing environmental textual information [2], firms may disclose additional environmental philosophies and slogans to construct a favorable environmental image [46]. Such disclosure enables them to obtain financing convenience, preferential policy treatment, and reputational gains [47,48]. Consequently, corporate managers are incentivized to manipulate CSR reports and embellish environmental textual information. Based on the above reasoning, we propose Hypothesis H1.

**H1:** There is an embellishment component in the environmental text information disclosed by companies, and companies engage in environmental information embellishment behaviors.

### 2.2. Impact of excessive environmental text disclosure on stock price crashes

In 2024, the Public Environmental Research Center released over 3.2 million environmental regulatory records, many of which required companies to rectify their practices within a specific timeframe. Companies that have been penalized for environmental violations tend to engage in proactive environmental text disclosure to maintain their public image. Under environmental regulations, most companies, driven by various motivations, tend to disclose environmental information positively. Some even engage in excessive greenwashing. Even though management may be aware that such actions could reduce analysts’ attention to the company [49], increase investors’ irrational investment behavior [50], and raise the cost of bond financing and financing constraints, they still continue with greenwashing, particularly companies that have received environmental penalties.

As environmental protection concepts deepen, environmental information has become an important indicator for investors to assess company value [34] Based on behavioral economics theory, investors tend to pay particular attention to information that deviates from expectations [51]. As negative news continues to accumulate, the increasing deviation from expectations can trigger panic in investor sentiment [52], ultimately leading to sharp fluctuations in stock prices [45]. Since China’s stock market is primarily composed of retail investors, only a small proportion of investors can recognize environmental text embellishment behaviors [2]. Even if they can identify such behaviors, they may be unable to confirm the reliability of the CSR reports. Moreover, environmental embellishment can be hidden in the short term without triggering immediate risks for the company. However, as embellished information accumulates over time, and as analysts, the public, and regulatory agencies dig deeper, the company’s true environmental situation will eventually be exposed. This will ultimately lead to a decline in corporate earnings and an increase in long-term risks [43]. Based on the above, we propose the following hypothesis:

**H2:** Excessive environmental text disclosure increases the risk of stock price crashes.

## 3. Research design

Based on the theoretical analysis presented earlier, this study first verifies the presence of greenwashing components in companies’ environmental text disclosures and then examines whether excessive environmental text disclosure exacerbates the risk of stock price crashes.

### 3.1. Data sources and methodology

The sample consists of Chinese A-share listed firms that issued CSR reports between 2015 and 2023. Firms in the financial sector and those designated as ST or *ST were excluded. In addition, reports presented primarily in image format, observations with missing key variables, and extreme values were removed. Continuous variables were winsorized at the 1% level at both tails. After screening and cleaning, the final dataset comprises 6,966 firm-year observations from 1,053 firms, derived from 10,899 valid CSR reports. CSR reports were collected from the Cninfo database using Python-based web crawlers, while supplementary data were obtained from the WIND and CSMAR databases (Shenzhen Stock Exchange Information Disclosure Platform (cninfo.com): http://www.cninfo.com.cn; CSMAR Database (China Stock Market & Accounting Research Database): https://www.gtarsc.com/; WIND Database: https://www.wind.com.cn/). All data processing and statistical analyses were conducted using Stata and SPSS. The methods of data collection and analysis are in compliance with the terms and conditions of Python web crawler technology.

For the empirical analysis, we adopt threshold effect models and quantile regression models. The threshold effect model is well-suited to capture potential nonlinearities in the relationship between environmental disclosure and stock price crash risk, whereas quantile regression allows for the estimation of effects across different points of the conditional distribution. This approach enhances the robustness of the results by reducing biases that may arise from a small number of exceptional firms deviating from mainstream patterns.

### 3.2. Model specification

In the first step, we verify the presence of greenwashing components in the environmental text disclosures. We assess the environmental text information against substantial environmental information. Following the approaches of Hansen [53] and Zhang et al. [43], we establish single, double, and triple threshold models to test the impact of environmental text disclosure on substantive disclosures. The model is specified as follows Model (1):


$$mr_{i,t}=\alpha_{0}+\alpha_{11}mw_{i,t}\times I(mw_{i,t}\leq h_{1})+\alpha_{12}mw_{i,t}\times I(mw_{i,t}>h_{1})+\alpha_{2}X_{i,t}+\lambda_{t}+\mu_{r}+\tau_{j}+\varepsilon_{i,t}$$ (1)

In the model, i denotes the firm, and t represents the year. $mr_{i,t}$ and $mw_{i,t}$ indicate the substantive disclosure volume and textual disclosure volume, respectively. $X_{i,t}$ refers to the control variables, while $\tau_{j}$, $\mu_{r}$, $\lambda_{t}$ and $\varepsilon_{i,t}$ correspond to the industry fixed effects, provincial fixed effects, year fixed effects, and the random disturbance term, respectively. I (·) is the indicator function, which equals 1 when the threshold variable meets the condition specified by $h_{1}$, and 0 otherwise. $\alpha_{11}$ and $\alpha_{12}$ represent the estimated coefficients when the textual disclosure volume $mw_{i,t}$ reaches the threshold, which reflects the impact on substantive disclosure volume. As the volume of textual disclosure increases, the growth rate of substantive disclosure volume is slower than that of textual disclosure, suggesting that the textual disclosure may contain “embellished” elements.

At the same time, a quantile regression model is employed to divide the substantive environmental information disclosure into poorer and better samples. If, in the poorer sample, the impact of textual disclosure on substantive disclosure is significantly lower, it suggests that the textual information disclosure contains embellishment elements. Specifically, if the value of $\beta_{1q}$ at the 20th percentile is much lower than that at the 80th percentile, it indicates the presence of embellishment in the environmental textual information. As shown in Model (2):


$$Q_{q}(mr_{i,t})=\beta_{0q}+\beta_{1q}mw_{i,t}+\beta_{2q}X_{i,t}+\lambda_{t}+\mu_{r}+\tau_{j}+\varepsilon_{i,t}$$ (2)

In this context, q represents the quantile of the substantive disclosure volume, while $\beta_{1q}$ denotes the impact of textual disclosure on substantive disclosure. The meanings of other variables remain consistent with Model (1).

The second step is to verify whether excessive environmental textual information disclosure exacerbates the risk of stock price crashes. First, the volume of environmental textual information disclosure is divided into excessive environmental textual disclosure (mw-above) and effective environmental textual disclosure (mw-below) based on the median. Model (3) is then constructed as follows:


$$cash_{i,t}=\gamma_{0}+\gamma_{1}mw\_above_{i,t}+\gamma_{2}X_{i,t}+\lambda_{t}+\mu_{t}+\tau_{j}+\varepsilon_{i,t}$$ (3)

$$cash_{i,t}=\gamma_{0}+\gamma_{1}mw\_below_{i,t}+\gamma_{2}X_{i,t}+\lambda_{t}+\mu_{t}+\tau_{j}+\varepsilon_{i,t}$$ (4)

Here, cash represents the stock price crash variable, specifically the negative skewness of returns (NCSKEW_i,t_) and the ratio of return volatility (DUVOL_i,t_). mw−above and mw−below refer to the excessive environmental text disclosure and the effective environmental text disclosure, respectively. Other variables are consistent with those in Model (1).

### 3.3. Measurement of variables

#### 3.3.1. Environmental text disclosure.

Following the approach of Zhang et al. [43], Li and Li [46], and Sun et al. [2], the social responsibility report is used as a source for extracting environmental text information. First, social responsibility reports were crawled from the Cninfo website using Python, converted into text files, and then segmented using Jieba for precise word segmentation. Words such as modal particles and those of length one were removed. A custom dictionary was created based on the keywords from Zhang et al. [43]. For each enterprise, the number of occurrences (num) of each keyword in their annual social responsibility report was counted, and the total number of keywords (totalnumber) was calculated for each year. The text disclosure amount (mw) was defined as $mw=\frac{num}{totalnumber}$. Following the substantive environmental information disclosure index system established by Zhang et al. [43] (see Table 1), the substantive disclosure score (mr) was manually organized in Excel, with a range of [0,36]. To achieve scale comparability between textual disclosure (mw) and substantive disclosure (mr), both variables were standardized using the Z-score method. The subsequent analysis relies on the differences between the standardized values of mw and mr to examine whether firms engage in greenwashing behavior, manifested as a “decoupling between rhetoric and action.”

**Table 1:** Substantive environmental information indicator system.

Category	Substantive Environmental Information Indicator	Rating Criteria
Reliability	Whether the Corporate Social Responsibility (CSR) Report is Prepared in Accordance with GRI Standards^1^	Reference to GRI Standards: 1 for Yes, 0 for No
	ISO 14001 Certification	Certified: 1 for Yes, 0 for No
	Whether the Auditor is from a Big Four Accounting Firm: 1 for Yes, 0 for No	Is from a Big Four Accounting Firm: 1 for Yes, 0 for No
	Whether the Report has been Verified by a Third-Party Organization: 1 for Yes, 0 for No	Verified by Third-Party: 1 for Yes, 0 for No
	Honors or Awards Received by the Company for Environmental Protection	Received: 1 for Yes, 0 for No
Specificity	Compliance with Pollutant Emission Standards	Disclosed: 1 for Yes, 0 for No
	Safety Production Status	Disclosed: 1 for Yes, 0 for No
	Disclosure of Environmental Negative Events (3 Categories): Sudden Environmental Accidents, Environmental Violations, Environmental Petition Cases	Disclosed: 1 for Yes, 0 for No. Value Range: [0, 3]
	Environmental Protection Initiatives and Other Social Welfare Activities Participated in by the Company	Disclosed: 1 for Yes, 0 for No
	The Company Has Established an Emergency Mechanism for Major Environmental Incidents	Disclosed: 1 for Yes, 0 for No
	“Three Simultaneities” System	Implemented: 1 for Yes, 0 for No
	Implementation of Cleaner Production	Disclosed: 1 for Yes, 0 for No
	Disclosure of Pollutant Emissions (6 categories): Wastewater, COD, SO2, CO2, Smoke and Dust, Industrial Solid Waste	No Disclosure: 0, Qualitative Disclosure: 1, Quantitative Disclosure: 2. Value Range: [0, 12]
	Disclosure of Pollutant Treatment (5 categories): Air Emissions, Wastewater, Smoke and Dust, Noise and Light Pollution, Solid Waste	No Disclosure: 0, Qualitative Disclosure: 1, Quantitative Disclosure: 2. Value Range: [0, 10]

Note: ^1^GRI (Global Reporting Initiative) is responsible for developing sustainability reporting standards. The report’s adherence to GRI guidelines indicates its compliance with established reporting norms.

#### 3.3.2. Risk of stock price collapse.

Following the approach of Chen et al. [54] and Hutton et al. [21], this study selects the negative skewness coefficient of returns (NCSKEW) and the ratio of upper to lower return volatility (DUVOL) as measures of stock price crash risk. The risk of stock price collapse is calculated using the following method:

First, the weekly data of individual stock i is used annually to perform a regression of Model (4). The residuals are then used to compute the stock-specific weekly return, W_ik_.


$$r_{ik}=\alpha_{i}+\beta_{1}r_{M,k-2}+\beta_{2}r_{M,k-1}+\beta_{3}r_{k}+\beta_{2}r_{M,k+1}+\beta_{2}r_{M,k+2}+\varepsilon_{i,k}$$ (5)

Where $r_{i,k}$ is the cash dividend-adjusted return for individual stock i in week k; $r_{M,k}$ is the market’s value-weighted average weekly return of all stocks in week k; to exclude the effects of asynchronous trading, the lagged and lead terms of the market return $r_{M,k}$ are included in Model (1) and $\varepsilon_{i,k}$ represents the residual term. The stock-specific weekly return is $W_{i,k}=Ln(1+\varepsilon_{i,k})$.

Next, based on W_i, k_a measure of stock price crash risk is constructed.

(1) Negative Skewness Coefficient of Returns (NCSKEW):

$$NCSKEW_{i,t}=-\frac{\left[n{\left(n-\text{1}\right)}^{3/2}\sum {W^{\text{3}}}_{i,k}\right]}{\left[\left(n-\text{1}\right)\;\left(n-\text{2}\right)\;{\left(\sum {W^{\text{2}}}_{i,k}\right)}^{3/2}\right]}$$ (6)

(2) Return Volatility Ratio (DUVOL):

$$DUVOL_{i,t}=log\frac{\left[\left(n_{u}-\text{1}\right)\sum_{down}{W^{\text{2}}}_{i,k}\right]}{\left[\left(n_{d}-\text{1}\right)\sum_{up}{W^{\text{2}}}_{i,k}\right]}$$ (7)

In Model (5), n represents the number of trading weeks for stock i in a given year. In Model (6), n_d_ and n_u_ denote the number of weeks in which the weekly return of stock i is below or above the annual average return, respectively.

#### 3.3.3. Control variables.

Building on the methodologies of Zhang et al. [43], Li and Li [46], Cai et al. [55], and Gong [56], this study controls for the following variables: monthly average excess turnover rate (dturn), standard deviation of the company’s annual weekly returns (sigma), stock’s annual weekly returns (returnt), debt-to-equity ratio (lev), company size (size), growth potential (growth), ownership concentration (ocr), executive compensation (ec), company age (age), independent directors (idr), ownership structure (se), managerial ownership (msr), and dual roles in management (tjc). The definitions of the relevant variables are provided in Table 2.

**Table 2:** Variable definitions.

Type	Variable	Name	Definition
**Dependent Variable**	Negative Skewness Coefficient	NCSKEW	Calculated as shown in Model (5)
	Return Volatility Ratio	DUVOL	Calculated as shown in Model (6)
**Independent Variable**	Excessive Environmental Text Disclosure	mw_above	Values greater than the median of the text disclosure amount
	Effective Environmental Text Disclosure	mw_below	Values less than the median of the text disclosure amount
**Control variables**	Monthly Average Excess Turnover Rate	dturn	The difference between the monthly turnover rate of stock i in month t and the previous month _t − 1_
	Standard Deviation of Annual Weekly Returns	sigma	The annual standard deviation of the specific weekly return of stock i in year t
	Annual Weekly Returns	returnt	The average weekly return of stock i in year t
	Debt-to-Equity Ratio	lev	The total liabilities divided by total assets
	Company Size	size	The natural logarithm of the company’s total assets
	Growth Potential	growth	The growth rate of the company’s main business revenue
	Ownership Concentration	ocr	The ownership percentage of the fifth largest shareholder
	Executive Compensation	ec	The average compensation of the Top 3 executives (log-transformed)
	Company Age	age	The number of years since the company was established
	Independent Directors	idr	The ratio of independent directors to total board members
	Ownership Structure	se	1 for state-owned enterprises, 0 for non-state-owned enterprises
	Managerial Ownership	msr	The percentage of shares owned by the company’s management
	Dual Role in Management	tjc	1 if the CEO and chairman are the same person, 0 otherwise
**Other Variables**	Environmental Text Disclosure Frequency	Mw	The ratio of environmental text word frequency to total report word frequency
	Substantive Disclosure Amount	Mr	The indicator system calculated based on Table 1

## 4. Empirical analysis

### 4.1. Descriptive statistics

After data processing, the descriptive statistics of the key variables are presented in Table 3. The mean values of **NCSKEW** and **DUVOL** are −0.105 and 0.219, respectively, with the minimum and maximum values being −1.276 and 0.843, and −2.652 and 3.396, respectively. These results indicate considerable differences in the risk and return profiles of the sample companies. The **Excessive Environmental Text Disclosure (mw-above)** has a mean of 1.36, with minimum and maximum values of 0.0195 and 5.361. The **Effective Text Disclosure (mw-below)** has a mean of 1.351, with minimum and maximum values of 0.502 and 3.694. It can be observed that only about 30% of the companies engage in excessive environmental information disclosure, while 70% of the companies engage in effective disclosure. The statistical values of other variables have been tested and are reasonably distributed, as shown in Table 3.

**Table 3:** Descriptive statistics.

Variable	N	Mean	Standard Deviation	Minimum	Maximum
ncskew	6966	−0.105	0.188	−1.276	0.843
duvol	6966	0.219	0.742	−2.652	3.396
Mw	6966	3.879	1.555	0.502	9.05674
Mw-above	3483	1.360	1.232	0.0195	5.361
Mw-below	3483	2.702	0.7408	0.502	3.694
Mr	6,966	10.356	6.310	1	31
Sigma	6966	0.061	0.010	0.050	0.083
dturn	6966	−29.206	402.796	−436.916	376.135
lev	6966	30.762	26.859	0.030	93.813
lnsize	6966	17.288	4.715	11.136	28.697
gro	6966	8.359	22.140	−51.408	133.439
ocr	6966	53.374	16.214	10.446	94.917
ec	6966	7.689	6.173	0.390	21.586
age	6966	22.083	5.920	4.000	65.000
idr	6966	37.944	6.925	16.670	66.670
returnt	6966	3.498747	28.99035	−33.9506	58.7476
msr	6966	4.849	11.195	0.000	77.350
se	6966	0.511	0.500	0.000	1.000
tjc	6966	0.226	0.419	0.000	1.000

### 4.2. Environmental text disclosures contain cosmetic elements

If an increase in the volume of environmental text information disclosure leads to a weaker explanatory effect on substantive disclosure, it suggests the presence of greenwashing in environmental information disclosure. To examine the nonlinear impact of text disclosure volume on substantive disclosure, this study employs a threshold effect model, using 300 rounds of Bootstrap sampling. The results, as presented in Table 4 (1)-(3), indicate the existence of significant single, double, and triple threshold effects, providing the basis for further empirical analysis.

**Table 4:** Impact of environmental text disclosure volume on substantive environmental information disclosure.

Variable Name	(1)	(2)	(3)	(4)	(5)
	Threshold Regression			Quantile Regression	
	Mr	Mr	Mr	20th Percentile	80th Percentile
	Single Threshold	Double Threshold	Triple Threshold		
Mw				1.448***	1.829***
				(38.8)	(19.36)
mw ≤ q_1_	0.880***	3.369***	3.536***		
	(14.392)	(5.111)	(5.364)		
q_1 < _mw or q_1_ ≤ mw ≤ q_2_	0.798***	0.927***	0.981***		
	(15.303)	(14.877)	(15.443)		
q_2 < _mw or q_2_ ≤ mw ≤ q_3_		0.832***	0.934***		
		(15.743)	(16.066)		
q_3 < _mw			0.732***		
			(12.643)		
Single Threshold	6.694	0.9518	8.2139		
Double Threshold		6.694	0.9518		
Triple Threshold			6.694		
F	33.91	32.36	31.32		
P	0.00	0.00	0.00		
R-squared	0.066	0.069	0.072		
Control Variables	Control	Control	Control	Control	Control

Note: t-statistics in parentheses*** p < 0.01, ** p < 0.05, * p < 0.1.

From the single threshold results in Table 4 (1), it is evident that as text disclosure volume increases, its explanatory power over substantive disclosure decreases (0.880 > 0.798). The double and triple threshold estimates in Table 4 (2)-(3) further confirm that the explanatory effect of text disclosure volume on substantive disclosure continues to weaken. To further validate this conclusion, we selected a subsample where the text disclosure volume is below the threshold value of 0.9518 and calculated the mean substantive disclosure volume (mr). The mean value of this subsample is 7.6276, which is lower than the overall sample mean (10.356). This suggests that some companies disclose environmental text information even in the absence of substantive environmental information, confirming the presence of greenwashing behavior. These empirical results are consistent with the findings of Zhang et al. [43].

Additionally, we employ a quantile regression model to examine the relationship between text disclosure volume and substantive disclosure. By estimating coefficients at the 20th and 80th quantiles of substantive disclosure, we find that, under the same level of environmental text information disclosure, firms with lower substantive disclosure exhibit a significantly weaker effect of text disclosure on substantive disclosure compared to firms with higher substantive disclosure. This indicates the presence of greenwashing behavior. As shown in Table 4 (4)-(5), in the sample with poor substantive disclosure (20th quantile), the effect of text disclosure volume on substantive disclosure is significantly lower (1.448 < 1.829), further confirming the existence of greenwashing in environmental information disclosure.

### 4.3. Verifying the impact of excessive environmental textual disclosure on stock price crash

To examine the impact of excessive environmental text information disclosure on stock price crashes, we test Hypothesis H2: If excessive environmental greenwashing is significantly positively correlated with stock price crashes, then H2 is confirmed. The regression results, as shown in Table 5 (1)-(2), indicate that the volume of excessive environmental text information disclosure (mw-above) is positively correlated with stock price crashes and is significant at the 10% level. This suggests that excessive disclosure of environmental text information exacerbates the risk of stock price crashes. Meanwhile, the study also finds that effective environmental text information disclosure significantly mitigates the risk of stock price crashes, as shown in the regression results in Table 5 (3)-(4).

**Table 5:** Excessive environmental greenwashing increases the risk of stock price crashes.

	(1)	(2)	(3)	(4)
Variables	Ncskew	Duvol	Ncskew	Duvol
Mw-above	0.010*	0.013*		
	(1.660)	(1.759)		
Mw-below			−0.007**	−0.009*
			(−2.213)	(−1.747)
Constant	−0.211***	−1.297***	−0.200***	−1.283***
	(−4.648)	(−14.461)	(−4.403)	(−14.220)
R-squared	0.027	0.267	0.027	0.267
Control Variables	Control	Control	Control	Control
Fixed Effects	Control	Control	Control	Control

Note: t-statistics in parentheses*** p < 0.01, ** p < 0.05, * p < 0.1.

### 4.4. Robustness test

#### 4.4.1. Instrumental variables approach.

To address potential endogeneity concerns, we employ an instrumental variable (IV) estimation. Given the lagged nature of environmental textual disclosure, the one-period lag of disclosure volume is adopted as the instrument to test Hypothesis H2. The results, reported in Columns (1)-(2) of Table 6, indicate that excessive environmental textual disclosure significantly increases the likelihood of stock price crashes, with the coefficients being highly significant at the 1% level. This provides strong support for Hypothesis H2. In contrast, Columns (3)-(4) show the effects of effective environmental textual disclosure. While the results are statistically insignificant, the coefficients are negative, suggesting that effective disclosure tends to alleviate the risk of stock price crashes.

**Table 6:** Robustness test.

Variables	Instrumental Variables Method				Alternative Measurement of the Dependent Variable	
	(1)	(2)	(3)	(4)	(5)	(6)
	ncskew	duvol	ncskew	duvol	synzonghe	synzonghe
mw_above	0.010***	0.031***			0.008***	
	(3.512)	(3.104)			(3.505)	
mw_below			−0.009***	−0.008		−0.000
			(−3.133)	(−0.814)		(−0.007)
Constant	−0.211***	−1.292***	−0.191***	−1.315***	0.222***	0.221***
	(−7.322)	(−13.290)	(−6.514)	(−13.331)	(7.201)	(5.481)
R-squared	0.027	0.267	0.025	0.266	0.112	0.111
Control Variables	Control	Control	Control	Control	Control	Control
Fixed Effects	Control	Control	Control	Control	Control	Control

Note: Robust z-statistics in parentheses*** p < 0.01, ** p < 0.05, * p < 0.1.

#### 4.4.2. Alternative measurement of the dependent variable.

Drawing on the research of Zhang et al. [57], stock price crashes are strongly positively correlated with stock price synchronicity. The higher the stock price synchronicity, the more pronounced the “co-movement” phenomenon, indicating that the company-specific information contained in stock price fluctuations is lower. There is a greater chance that bad news will be concealed, which in turn increases the risk of a stock price crash.

First, following the approach of Durnev et al. [58] and Jin and Myers [59], regression estimation is performed using Equation (8), with the goodness-of-fit (R²) of Model (7) used to measure stock price synchronicity. Since the goodness-of-fit value ranges from 0 to 1 and does not follow a normal distribution, it is necessary to adjust it using Equation (9).


$$R_{i,t}=\beta_{\text{0}}+\beta_{\text{1}}*R_{m,t}+\beta_{\text{2}}*R_{I,t}+\varepsilon$$ (8)

$$Synch=ln\frac{R^{\text{2}}}{1-R^{\text{2}}}$$ (9)

The regression results, as shown in Table 6 (5), indicate that excessive environmental text information disclosure is positively correlated with stock price crashes at the 1% significance level, supporting the original hypothesis. Table 6 (6) shows the impact of effective environmental text information disclosure on stock price crashes. Although not statistically significant, the effect is in the opposite direction, suggesting a certain suppressive effect.

#### 4.4.3. Placebo test.

**Fig 1:**
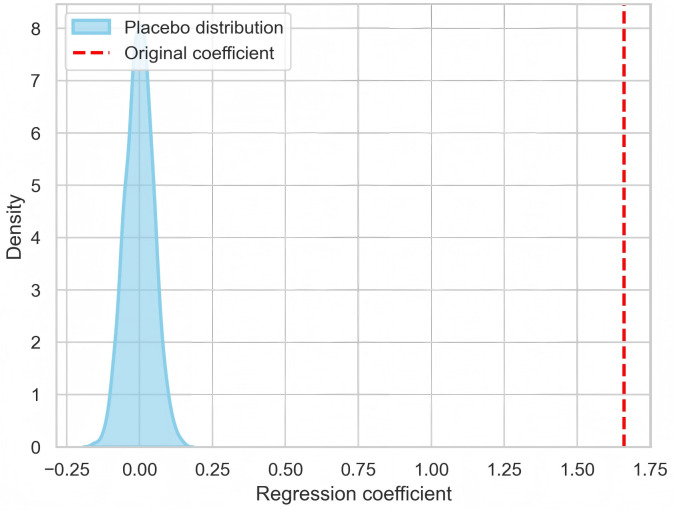
Placebo test of the association between substantive disclosure and textual disclosure.

To mitigate concerns that the regression results between textual disclosure (mw) and substantive disclosure (mr) may arise from randomness, we randomly shuffled the pairings between environmental textual disclosure (mw) and substantive disclosure (mr). The results show that the distribution of the placebo coefficients approximately follows a normal distribution centered around zero, whereas the true estimated coefficient lies far outside this distribution (see Fig 1). This finding indicates that our results are not driven by random matching, thereby supporting the validity of our greenwashing identification.

## 5. Further analysis

### 5.1. Mechanism test

The mechanism through which excessive environmental text information disclosure leads to the risk of stock price crashes is as follows: the excessive greenwashing of environmental information exacerbates information asymmetry, reduces the quality of information disclosure, and intensifies irrational investor sentiment. Once negative news reaches a certain threshold, it will inevitably be fully released, manifesting in the capital markets as a stock price “Waterloo” (stock price crash). This paper uses the information disclosure quality rating (infq) provided by the Shanghai and Shenzhen Stock Exchanges to measure information transparency. If there is a significant negative correlation between the volume of excessive environmental text information disclosure and information transparency (infq), it will confirm that this transmission mechanism is correct. Drawing on the studies of Cheng and Chen [60] and Liu et al. [61], this study employs the Tobin’s Q decomposition model for annual and industry-specific regressions. The residuals (E) from this model measure the irrational sentiment of investors at the firm level (sent), as shown in Model 9


$$Q_{i,t}=\delta_{0}+\delta_{1}ROE_{i,t}+\delta_{2}GRO_{{}_{i,t}}+\delta_{3}LEV_{i,t}+\delta_{4}SIZE_{{}_{i,t}}+\varepsilon_{{}_{i,t}}$$ (10)

$Q_{i,t}$ represents the Tobin’s Q value of firm i at the end of year t;

$ROE_{i,t}$ is the return on equity of firm i in year t;

$GRO_{i,t}$ is the revenue growth rate of firm i in year t;

$LEV_{i,t}$ is the leverage ratio of firm i at the end of year t;

$SIZE_{i,t}$ is the logarithm of total assets of firm i at the end of year t.

If the volume of excessive environmental text information disclosure is significantly positively correlated with investor sentiment, it suggests that excessive greenwashing of text information exacerbates irrational investor sentiment. The regression results from the above validation, as shown in Table 7 (1) and (3), indicate that excessive environmental text information disclosure is significantly negatively correlated with information transparency at the 1% level and positively correlated with investor sentiment at the 5% level. That is, excessive environmental text information disclosure reduces information transparency and amplifies investors’ irrational investment sentiment.

**Table 7:** Mechanism test—information transparency and investor sentiment.

	(1) Information Transparency	(2) Information Transparency	(3) Investor Sentiment	(4) Investor Sentiment
VARIABLES	infq	infq	sent	sent
mw_above	−0.045***		0.004**	
	(−3.304)		(1.994)	
mw_below		0.052***		−0.002
		(5.031)		(−1.414)
Constant	0.866***	0.782***	1.946***	1.949***
	(4.442)	(4.002)	(62.914)	(62.756)
R-squared	0.369	0.371	0.793	0.793

Note: t-statistics in parentheses*** p < 0.01, ** p < 0.05, * p < 0.1.

Furthermore, the study found that effective environmental text information disclosure enhances the quality of information disclosure and curbs irrational investor sentiment, as shown in Table 7 (2) and (4). These estimated results support the hypothesized outcomes, indicating that excessive environmental information disclosure diminishes the quality of information disclosure, exacerbates irrational investor sentiment, and accelerates stock price crashes.

### 5.2. Heterogeneity test

From a mechanistic perspective, the manufacturing sector serves not only as the backbone of the national economy but also as a major source of energy consumption and pollutant emissions. Consequently, it is subject to stricter environmental regulations and greater social scrutiny, and investors place higher emphasis on the quality of its environmental disclosures [62]. Moreover, Made in China 2025 underscores the central role of manufacturing in the modernization process, while also highlighting its critical function in the green transition. According to signaling theory, when firms convey a “responsible” signal through environmental responsibility disclosure, investors are more likely to form positive expectations and to accept higher environmental investment risks [63,64]. At the same time, pressures from green financing [65,66] and regulatory compliance further incentivize manufacturing firms to excessively embellish environmental information in order to alleviate external pressures. Based on these considerations, this study classifies industries following the Guidelines on Industry Classification of Listed Companies issued by the China Securities Regulatory Commission (2012 edition, last revised in 2023), dividing firms into manufacturing and non-manufacturing groups, and examines the relationship between excessive environmental textual disclosure and stock price crash risk across different industries. The regression results are presented in Table 8 (1)-(4). The study finds that, in manufacturing enterprises, excessive environmental information disclosure significantly increases the risk of stock price crashes at a 5% significance level, while in non-manufacturing industries, the regression results are not significant. The regression results in Table 8 (5)-(8) show that, in manufacturing enterprises, effective environmental information disclosure can suppress stock price crashes at a 10% significance level. Although, in non-manufacturing enterprises, effective environmental information disclosure is not significantly correlated with stock price crashes, it still shows a negative correlation, exerting a certain suppressive effect.

**Table 8:** Heterogeneity test.

	Manufacturing Industry		Non-Manufacturing Industry		Manufacturing Industry		Non-Manufacturing Industry	
	(1)	(2)	(3)	(4)	(5)	(6)	(7)	(8)
VARIABLES	ncskew	duvol	ncskew	duvol	ncskew	duvol	ncskew	duvol
mw_above	0.009***	0.022**	0.010***	−0.000				
	(3.088)	(2.348)	(3.643)	(−0.027)				
mw_below					−0.013***	−0.015*	−0.001	−0.004
					(−5.024)	(−1.883)	(−0.311)	(−0.530)
Constant	−0.381***	−1.402***	−0.094**	−1.225***	−0.359***	−1.378***	−0.094**	−1.218***
	(−8.480)	(−9.926)	(−2.541)	(−8.979)	(−7.978)	(−9.703)	(−2.509)	(−8.888)
Observations	3,144	3,144	3,533	3,533	3,144	3,144	3,533	3,533
R-squared	0.046	0.249	0.030	0.286	0.051	0.249	0.027	0.286

Norwe: t-statistics in parentheses*** p < 0.01, ** p < 0.05, * p < 0.1.

### 5.3. Governance pathways

Based on the aforementioned mechanism tests, this paper further explores the governance pathways to curb excessive environmental text information disclosure by enterprises. The study examines how shareholder structure and corporate governance mechanisms can inhibit such behavior. Research by Lin and Chu [67] and Shen et al. [68] found that large shareholders tend to conceal unreasonable related-party transactions, exacerbating the agency problems between large and small shareholders, which can lower information transparency. From the perspective of ownership concentration, the regression results shown in Table 9 (1)-(2) indicate that higher ownership concentration significantly amplifies the behavior of excessive environmental text information disclosure at the 10% significance level. That is, the higher the ownership concentration, the more motivated a company is to embellish environmental information. Therefore, reducing ownership concentration is necessary to curb excessive environmental information disclosure. The relationship between ownership concentration and effective information disclosure is positive but not statistically significant.

**Table 9:** Governance pathways—shareholder structure and corporate governance.

	(1)	(2)	(3)	(4)	(5)	(6)
VARIABLES	Mw-above	Mw-below	Mw-above	Mw-below	Mw-above	Mw-below
ocr	0.002**	0.001				
	(2.151)	(0.375)				
idr			−0.008***	0.011***		
			(−3.870)	(4.221)		
msr					−0.003**	0.003*
					(−2.384)	(1.899)
Constant	−0.367**	2.109***	−0.011	1.726***	−0.270*	2.082***
	(−2.413)	(10.521)	(−0.065)	(7.969)	(−1.805)	(10.559)
R-squared	0.036	0.016	0.038	0.019	0.037	0.015

Note: t-statistics in parentheses*** p < 0.01, ** p < 0.05, * p < 0.1.

The independent director system refers to the introduction of third-party directors, who have no ties to the company’s existing management, to supervise and reduce insider control, thereby safeguarding fairness and protecting the interests of minority shareholders. Independent directors, constrained by reputation mechanisms, are likely to exercise more effective supervision over the management [69]. The regression results in Table 9 (3)-(4) show that the proportion of independent directors significantly inhibits excessive environmental text information disclosure by management and promotes effective environmental information disclosure. Therefore, it is crucial to strengthen the role and position of independent directors within the formal system, granting them the corresponding functions and powers to effectively reduce the embellishment of environmental text information by enterprises.

Based on the incentive effects of equity-based compensation, some scholars have found that management ownership can significantly alleviate agency problems and managerial myopia, encouraging management to actively participate in corporate operations and improving the quality of information disclosure, financial performance [70], corporate value [71], and the effectiveness of internal controls [72]. From the perspective of management ownership, the regression results in Table 9 (5)-(6) indicate that management ownership significantly suppresses excessive environmental text information disclosure. Furthermore, it was found that management ownership is positively correlated with effective information disclosure at the 10% significance level. Therefore, companies should implement management stock ownership plans and grant appropriate ownership percentages to management, which can effectively curb stock price crashes.

## 6. Conclusion and implications

This study selects Chinese A-share listed companies that published social responsibility reports from 2015 to 2023 as the research sample. Through the threshold effect and quantile regression models, it verifies the existence of embellishments in the environmental textual information disclosed by firms. The disclosed environmental textual information is then categorized based on the median into two groups: excessive environmental textual disclosure and effective environmental textual disclosure. The study further investigates the potential impact of excessive environmental textual disclosure on stock price crashes.

The findings are as follows:

1. **Environmental Textual Information Contains Embellishments:** Both the threshold effect and quantile regression models support the notion that environmental textual information contains embellishments beyond substantial environmental data. In other words, the environmental textual information includes greenwashing elements.
2. **Impact of Excessive Environmental Textual Disclosure on Stock Price Crashes:** Excessive environmental textual disclosure exacerbates the risk of stock price crashes, while effective environmental textual disclosure mitigates the risk of stock price crashes. The mechanism is that excessive environmental textual disclosure reduces the quality of information disclosure and fuels irrational investor behavior.
3. **Heterogeneity Analysis:** Compared to non-manufacturing firms, excessive environmental textual disclosure significantly increases the risk of stock price crashes in manufacturing firms. Effective environmental textual disclosure mitigates the risk of stock price crashes in manufacturing firms.
4. **Governance Pathways Analysis:** To improve the quality of environmental textual information disclosure, firms should reduce ownership concentration, increase management stock ownership, and actively leverage independent directors in the corporate governance process.

Implications: In China’s capital market, which is primarily retail investor-based, information users should not only “listen to the words” but also “observe the actions,” avoiding being misled by flashy textual information. Investment in the manufacturing industry should be approached with greater caution. Regulatory authorities should encourage firms to have their social responsibility reports audited by independent third parties to enhance the authenticity and objectivity of environmental information.

This study has several limitations. First, the measurement of greenwashing relies on textual analysis of corporate disclosures, which may not fully capture the nuanced strategies of managerial disclosure behavior. Second, our sample is limited to Chinese A-share listed companies, so caution is needed when generalizing the findings to other countries. Future research could extend the sample to firms in different countries or adopt alternative measures of greenwashing to further enhance the external validity of the results.

## Supporting information

S1 Table Basic regression data and robustness regression data. (RAR)

## References

[1] Zhang Q, Yu Z, Kong D. The real effect of legal institutions: environmental courts and firm environmental protection expenditure. J Environ Econ Manag. 2019;98:102254. doi: 10.1016/j.jeem.2019.102254

[2] Sun X, Che T, Ma X. Catering behavior of firms’ carbon information disclosure: Identification, premium loss and mechanisms. China Ind Econ. 2023;(1):132–50.

[3] Li X, Hu Y, Shi W. Initial exploration of ESG report top-level system design in China. Securities Market Herald. 2022;(4):35–44.

[4] Li Z. Will cheap talk on environmental responsibility get punished? J World Econ. 2018;41(12):167–88.

[5] Dai NT, Du F, Young SM, Tang G. Seeking Legitimacy through CSR Reporting: Evidence from China. J Manag Account Res. 2016;30(1):1–29. doi: 10.2308/jmar-51627

[6] Wang H, Cao F, Ye K. Supervision or hollowing out: The shareholding ratio of major shareholders and stock price crash risk. J Manag World. 2015;(2):45–57.

[7] Jian G, Gao Y. The exit threat of non-controlling major shareholders, social responsibility fulfillment, and stock price crash risk. Fin Econ. 2023;(5):59–70.

[8] Jiang X, Xu N. Corporate over-investment and stock price crash risk. Fin Res. 2015;(8):141–58.

[9] Wang H, Cao F, Gao S, Li Z. Investor protection and stock price crash risk. Fin Trade Econ. 2014;(10):73–82.

[10] Yang D, Hu K, Zhu S, Lin F. Stock price crash risk and online investor relations management: from the perspective of crisis public relations. J Central Univ Fin Econ. 2022;(3):69–80.

[11] Xu N, Li X, Yuan Q, Chan KC. Excess perks and stock price crash risk: evidence from China. J Corpor Fin. 2014;25:419–34. doi: 10.1016/j.jcorpfin.2014.01.006

[12] Wu D, Zhan N. The impact of managerial overconfidence on stock price crash risk: The mediating role of mergers and acquisitions goodwill. Fin Econ Res. 2020;35(5):108–20.

[13] Ding Y. Managerial overconfidence, stock price crash risk, and dynamic adjustment of capital structure: An analysis of mediating effects based on dynamic panel data. J Southeast Univ. 2022;24(2):74–84.

[14] Wang S, Du S, Zhang Z, Zhao X. Can management statements reduce stock price crash risk? Shanghai Fin. 2021;(11):26–33.

[15] Zheng Y, Xue M, Ou P. Media background, independent directors, and stock price crash risk. Modern Fin. 2019;(12):84–95.

[16] Wu X, Guan W. Celebrity independent directors, management power, and stock price crash risk. Modern Fin. 2020;40(1):98–113.

[17] Dong HY. The geographical proximity of independent directors with a financial background and the risk of stock price crash. J Shanxi Univ Fin Econ. 2016;(3):113–24.

[18] Yi X, Xie ZL. Independent directors’ network position, institutional environment and stock price crash risk. Fin Account Mthly. 2019;(11):17–26.

[19] Dong G, Ma G, Liu S. Independent director compensation and stock price collapse: Inhibition or promotion-based on a financial background perspective. PLoS One. 2023;18(8):e0289986. doi: 10.1371/journal.pone.0289986 37561795 PMC10414673

[20] Ye K, Cao F, Wang H. Can internal control information disclosure reduce stock price crash risk? Fin Res. 2015;(2):192–206.

[21] Hutton AP, Marcus AJ, Tehranian H. Opaque financial reports, R2, and crash risk⋆. J Fin Econ. 2009;94(1):67–86. doi: 10.1016/j.jfineco.2008.10.003

[22] Huang R, Xie X, Zhou H. The “isomorphic” behavior of corporate greenwashing. China Popul Resour Environ. 2020;30(11):139–50.

[23] Li CF. Regional economic difference, enterprise organizational change, environmental accounting information disclosure: empirical data from the polluting enterprises of Shanghai in 2009. J Audit Econ. 2012;27(1):68–78.

[24] Zhu P, Guo WF. The influence of environmental information disclosure quality on green innovation. J Jishou Univ (Soc Sci Ed). 2022;43(6):92–101.

[25] Wang L, Li Z, Sha Y. Collaboration or collusion: Heterogeneous institutional co-ownership and corporate greenwashing behavior. Fin Econo Rev. 2023;(8):70–80.

[26] Lu Z, Lin Y, Li Y. Does corporate engagement in digital transformation influence greenwashing? Evidence from China. Fin Res Lett. 2023;58:104558. doi: 10.1016/j.frl.2023.104558

[27] Zhang D. Can environmental monitoring power transition curb corporate greenwashing behavior? J Econ Behav Organ. 2023;212:199–218. doi: 10.1016/j.jebo.2023.05.034

[28] Yang G, Du Y, Liu Y. Corporate operating performance, media attention, and environmental information disclosure. Econ Manage. 2020;(3):55–72.

[29] Zhang D. Does green finance really inhibit extreme hypocritical ESG risk? A greenwashing perspective exploration. Energy Econ. 2023;121:106688. doi: 10.1016/j.eneco.2023.106688

[30] Sun Y. Bank competition and firm greenwashing: evidence from China. Fin Res Lett. 2024;63:105279. doi: 10.1016/j.frl.2024.105279

[31] Zhou B, Wang Q. FinTech matters in sustainable finance: does it redistribute the supply of financial services? J Int Fin Marke Inst Money. 2024;91:101913. doi: 10.1016/j.intfin.2023.101913

[32] Shen H, Feng J. Public opinion supervision, government regulation, and corporate environmental information disclosure. Account Res. 2012;(2):72–8.

[33] Liu Y, Zhang J, Dai Y. Analyst following and greenwashing decision. Fin Res Lett. 2023;58:104510. doi: 10.1016/j.frl.2023.104510

[34] Tang Y, Ma W, Xia L. Quality of environmental information disclosure, internal control “level” and enterprise value ——empirical evidence from listed companies in heavy polluting industries. Account Res. 2021;(7):69–84.

[35] Li H, Liu Q, Li S, Fu S. Environmental, social, and governance information disclosure and corporate green innovation performance. Stat Res. 2022;39(12):38–54.

[36] Zhang ZH, Jiang Y. Green credit policy, corporate environmental information disclosure and stock price crash risk. Fin Account Monthly. 2023;44(1):92–101.

[37] Yu L, Zhang W, Bi X. Environmental policy uncertainty and corporate environmental information disclosure: evidence from changes in local environmental protection officials. J Shanghai Univ Fin Econ. 2020;22(2):35–50.

[38] Yao S, Zhou M. Trade-off in corporate environmental information disclosure under policy changes: government subsidies and risk avoidance. Fin Trade Res. 2017;28(7):99–110.

[39] Li D, Jia X, Xin L. A review and prospect of corporate greenwashing behavior. Foreign Econ Manag. 2015;37(12):86–96.

[40] Tian L, Wang K. The “concealment effect” of social responsibility information disclosure and stock price crash risk: Evidence from the Chinese stock market based on DID-PSM analysis. J Manag World. 2017;(11):146–57.

[41] Huang R, Chen W, Wang K. External financing needs, impression management, and corporate greenwashing. Comp Econ Soc Syst. 2019;(3):81–93.

[42] Xiao H, Zhang J, Li W. Research on the behaviors of pseudo-CSR. China Ind Econ. 2013;(6):109–21.

[43] Zhang D, Zhang J, Dong S. The potential impact of environmental information whitewashing behavior. World Econ. 2024;47(4):99–128.

[44] Christensen DM, Serafeim G, Sikochi A. Why is corporate virtue in the eye of the beholder? The case of ESG ratings. The Accounting Review. 2021;97(1):147–75. doi: 10.2308/tar-2019-0506

[45] Zhao C, Chen SH, Cao W. “Internet Plus” information disclosure: substantive statement or strategic manipulation——Evidence based on the risk of stock price crash. China Ind Econ. 2020;(3):174–92.

[46] Li S, Li Z. Research on the signaling effect of the idiosyncratic information content of CSR reports——An analysis based on NLP technology. China Ind Econ. 2023;(1):114–31.

[47] Shen H, Huang Z, Guo F. Confess or defense? A study on the relationship between environmental performance and environmental disclosure. Nankai Bus Rev. 2014;17(2):56–63, 73.

[48] Li X, Liang R, Li Y. Does ESG affect stock liquidity? Evidence from the dual perspectives of ESG ratings and rating disagreement. Stud Int Fin. 2023;(11):75–86.

[49] Liao J, Su D. Negative reputation of listed companies and analyst coverage: Pursuing or avoiding? Account Res. 2021;(8):38–53.

[50] Cen L, Wei KCJ, Yang L. Disagreement, underreaction, and stock returns. Manag Sci. 2017;63(4):1214–31. doi: 10.1287/mnsc.2015.2405

[51] Hu T, Chen R, Tu Z. Environmental information disclosure evaluation and market value: the influence of third-party institutions. J World Econ. 2022;45(11):150–76.

[52] Song X, Hu J, Li S. Corporate social responsibility disclosure and stock price crash risk: Based on information effect and reputation insurance effect. Fin Res. 2017;(4):161–75.

[53] Hansen BE. Threshold effects in non-dynamic panels: Estimation, testing, and inference. J Econometr. 1999;93(2):345–68. doi: 10.1016/s0304-4076(99)00025-1

[54] Chen J, Hong H, Stein JC. Forecasting crashes: trading volume, past returns, and conditional skewness in stock prices. J Fin Econ. 2001;61(3):345–81. doi: 10.1016/s0304-405x(01)00066-6

[55] Cai W, Pu Y, Xia T, Wei Q. Business group, short-term financing and long-term investment and business risk. J World Econ. 2023;46(4):192–219.

[56] Gong YF. Impacts of internal control deficiencies and its rectification on stock price crash risk. J Zhongnan Univ Econ Law. 2020;(1):37–45.

[57] Zhang J, Liu B, Shen H. Stock price synchronicity and stock price crash risk: Based on information asymmetry and corporate governance perspectives. Fin Sci. 2019;(4):13–25.

[58] Durnev A, Morck R, Yeung B. Value‐enhancing capital budgeting and firm‐specific stock return variation. J Fin. 2004;59(1):65–105. doi: 10.1111/j.1540-6261.2004.00627.x

[59] Jin L, Myers S. R2 around the world: New theory and new tests⋆. J Fin Econ. 2006;79(2):257–92. doi: 10.1016/j.jfineco.2004.11.003

[60] Cheng C, Chen Q. Policy uncertainty, investor sentiment, and major shareholder pledge. Forecasting. 2020;39(2):63–9.

[61] Liu JQ, Yin GY, Wu JH. Tone of corporate social responsibility report and asset mispricing. Account Res. 2022;(5):131–45.

[62] Bebbington J, Larrinaga C, Moneva JM. Corporate social reporting and reputation risk management. Account Audit Account J. 2008;21(3):337–61. doi: 10.1108/09513570810863932

[63] Li Z, Feng L. Corporate ESG performance and access to commercial credit. Fin Res. 2022;48(12):151–65.

[64] Guo Y, Hong Y. The impact mechanism of ESG information disclosure on financing constraints: Empirical evidence from A-share listed companies in China. J Harbin Univ Commerce (Social Sciences Edition). 2023;(3):87–99.

[65] Zhao S, Cui Z, Liang W. The effect of digital finance on the green innovation development of high energy-consuming enterprises. Sci Manage Res. 2023;(6):131–8.

[66] Wu X, Liu Y. Green credit, digital transformation, and sustainable development of heavily polluting enterprises. J Xi’an Univ Fin Econ. 2025;(8):1–13.

[67] Lin ZG, Chu JJ. Conduction effect of internal control on the ownership structure and earnings quality. Taxa Econ. 2012;(6):1–11.

[68] Shen HY, Wu XH, Wu SN. Controlling shareholder’s control right and stock price crash risk: “Benefit synergy” or “tunneling” effect? Bus Manag J. 2017;43(4):65–83.

[69] John K, Senbet LW. Corporate governance and board effectiveness. J Banking Fin. 1998;22(4):371–403. doi: 10.1016/s0378-4266(98)00005-3

[70] Shen LP, Huang Q. Employee equity incentive and enterprise value and innovation from endogenous perspective. Securities Market Herald. 2016;2016(4):27–34.

[71] Liu G, Wang J. Empirical research on corporate shareholding structure, incentive system, and performance. Econ Theory Econ Manag. 2000;(5):40–5.

[72] Lu D, Wang Y, Fu P. Do CEO incentives improve internal control effectiveness? Evidence from state-owned listed companies. Account Res. 2014;(6):66–72.

